# The relationship between brain oscillatory activity and therapeutic effectiveness of transcranial magnetic stimulation in the treatment of major depressive disorder

**DOI:** 10.3389/fnhum.2013.00037

**Published:** 2013-02-26

**Authors:** Andrew F. Leuchter, Ian A. Cook, Yi Jin, Bill Phillips

**Affiliations:** ^1^Department of Psychiatry and Biobehavioral Sciences, David Geffen School of Medicine, Semel Institute for Neuroscience and Human Behavior, University of California Los AngelesLos Angeles, CA, USA; ^2^NeoSync, Inc.Waltham, MA, USA; ^3^Department of Psychiatry and Human Behavior, University of CaliforniaIrvine, CA, USA

**Keywords:** rTMS, sTMS, major depressive disorder, individual alpha frequency, mechanism of action of TMS

## Abstract

Major depressive disorder (MDD) is marked by disturbances in brain functional connectivity. This connectivity is modulated by rhythmic oscillations of brain electrical activity, which enable coordinated functions across brain regions. Oscillatory activity plays a central role in regulating thinking and memory, mood, cerebral blood flow, and neurotransmitter levels, and restoration of normal oscillatory patterns is associated with effective treatment of MDD. Repetitive transcranial magnetic stimulation (rTMS) is a robust treatment for MDD, but the mechanism of action (MOA) of its benefits for mood disorders remains incompletely understood. Benefits of rTMS have been tied to enhanced neuroplasticity in specific brain pathways. We summarize here the evidence that rTMS entrains and resets thalamocortical oscillators, normalizes regulation and facilitates reemergence of intrinsic cerebral rhythms, and through this mechanism restores normal brain function. This entrainment and resetting may be a critical step in engendering neuroplastic changes and the antidepressant effects of rTMS. It may be possible to modify the method of rTMS administration to enhance this MOA and achieve better antidepressant effectiveness. We propose that rTMS can be administered: (1) synchronized to a patient's individual alpha frequency (IAF), or synchronized rTMS (sTMS); (2) as a low magnetic field strength sinusoidal waveform; and, (3) broadly to multiple brain areas simultaneously. We present here the theory and evidence indicating that these modifications could enhance the therapeutic effectiveness of rTMS for the treatment of MDD.

## Introduction

The technique of transcranial magnetic stimulation (TMS) has its roots in brain neurophysiology. The technique is based on Faraday's principles of electromagnetic induction, and was first reported as a method for inducing currents in the human brain in 1985 (Barker et al., 1985). The electric potential associated with this brain current can be sufficient to depolarize neurons in motor cortex and generate a motor evoked potential (MEP). TMS subsequently has been shown to have robust and reproducible effects on perception and cognition, which have been posited to occur through creation of “virtual lesions” (Pascual-Leone et al., 2000).

Repetitive pulses of TMS, or repetitive transcranial magnetic stimulation (rTMS), was first demonstrated to be an effective therapeutic modality for major depressive disorder (MDD) in the mid-1990s (George and Wassermann, 1994; George et al., 1995; Pascual-Leone et al., 1996) and, over the past two decades, it repeatedly has been demonstrated to have therapeutic benefit for MDD (George et al., 2000, 2010; Dannon and Grunhaus, 2001; Grunhaus et al., 2002, 2003; Prudic et al., 2004; O'Reardon et al., 2007; Higgins and George, 2009; Shutter, 2009; Slotema et al., 2010; Carpenter et al., 2012). The enduring effects of rTMS on mood are consistent with the observed effects of repetitive stimulation on brain function, which has been reported to last for minutes to days after the termination of stimulation and extend well beyond the site of stimulation, suggesting sustained effects on excitability and plasticity of neuronal circuits (Siebner and Rothwell, 2003).

Evidence suggests that the effectiveness of rTMS in treating MDD may be based upon the effects of the technique in altering brain oscillatory activity and connectivity. We review below evidence regarding abnormal brain neurophysiology in MDD, the neurophysiologic effects of TMS and rTMS, how these effects might be related to the therapeutic mechanism of action (MOA) of rTMS in MDD, and then finally propose a novel approach to rTMS treatment based upon an understanding of brain neurophysiology in MDD.

## Brain connectivity and oscillatory activity in MDD

MDD involves dysfunction in a number of cortical regions, such as dorsolateral prefrontal cortex (DLPFC) and anterior cingulate cortex (ACC), as well as deep gray matter structures, such as nuclei of the thalamus and hypothalamus. The illness is increasingly understood as a disorder of connectivity in brain networks linking these regions (Leuchter et al., 2012). Many of the mood and neurovegetative symptoms, as well as deficits in cognition and memory, have been hypothesized to arise from dysfunction in networks linking cortical and subcortical gray structures (Ottowitz et al., 2002; Savitz and Drevets, 2009). Functional magnetic resonance imaging (fMRI) studies of resting-state blood oxygen level-dependent (BOLD) signal in MDD have shown overall increases in functional connectivity in the default mode and other brain networks (Greicius et al., 2007; Grimm et al., 2009; Sheline et al., 2010; Zhou et al., 2010) with complex patterns of altered resting connectivity between some cortical and subcortical structures (Bluhm et al., 2009; Cullen et al., 2009; Veer et al., 2010; Hamilton et al., 2011). Neurophysiologic studies using magnetoencephalography (MEG) or quantitative electroencephalography (qEEG) also have shown increased functional connectivity, as indicated primarily by increased theta (4–8 Hz) and alpha band (8–12 Hz) amplitude, power, coherence, and other measures of synchronized brain activity (Henriques and Davidson, 1991; Bruder et al., 1997; Debener et al., 2000; Knott et al., 2001; Pizzagalli et al., 2005; Fingelkurts et al., 2007). Synchronized oscillations operate on a more rapidly shifting time scale than BOLD signals (Britz et al., 2010) and oscillatory activity measured by qEEG has been shown to elicit BOLD signal activations within resting state networks (Musso et al., 2010). These studies highlight how measures of oscillatory activity and blood flow represent “two sides of the same coin,” and are complementary indicators of brain network activity.

MDD has been conceptualized as a syndrome of “thalamocortical dysrhythmia,” marked by persistent resonance of rhythmic thalamocortical activity during the waking state; other illnesses in the family of thalamocortical dysrhythmias include central pain syndromes, some epilepsies, and Parkinson's Disease (Llinás et al., 1999; Bish et al., 2004; Llinás and Steriade, 2006; Walton et al., 2010; Schulman et al., 2011). Theta and alpha activity are produced by the cerebral cortex under the strong influence of thalamic pacemakers (Buzsáki and Draguhn, 2004). There are not distinct populations pacemaker cells for the two frequency bands. Overlapping thalamic cell populations generate alpha and theta rhythms (c.f., Hughes and Crunelli, 2005), with the predominant rhythm dependent upon their rate of firing, which is dependent upon the activity of voltage dependent (T-type) calcium channels (Perez-Reyes and Lory, 2006). In thalamocortical dysrhythmias, there appears to be hyperpolarization of thalamic relay cells, with consequent de-inactivation of T-channels and persistent low-threshold calcium spiking, and resultant monotonous resonant low-frequency oscillations (Llinás et al., 1999; Llinás and Steriade, 2006; Schulman et al., 2011). The increased oscillatory synchrony in patients with MDD and other thalamocortical dysrhythmias measured using MEG is pronounced in the theta frequency band, but also is prominent in the alpha band and across different frequency bands (Llinás et al., 1999; Schulman et al., 2011). This finding of greatly increased oscillatory synchrony using MEG in MDD is consistent with reports of increased thalamocortical connectivity using fMRI (Greicius et al., 2007) and increased coherence across the frequency spectrum on qEEG (Leuchter et al., 2012).

Increased oscillatory synchrony in MDD has been reported in multiple frequency bands across brain regions (Fingelkurts et al., 2006, 2007; Leuchter et al., 2012); most reports, however, have highlighted increased alpha band synchrony (Henriques and Davidson, 1991; Bruder et al., 1997; Debener et al., 2000; Knott et al., 2001; Pizzagalli et al., 2005; Seagrave et al., 2010). The most consistent findings have been local increases in power, and therefore local synchrony, although the literature is mixed as to specific locations where this has been reported. Disturbances in frontal or posterior alpha power symmetry commonly have been noted, but some studies have shown relatively greater local alpha power and synchrony over left rather than right anterior sites, others the opposite pattern, and still others find primarily posterior differences (Tucker et al., 1981; Davidson et al., 1987; Henriques and Davidson, 1991; Bruder et al., 1997; Jin et al., 1997; Gotlib, 1998; Coan and Allen, 2004). One report found that the pattern of frontal alpha asymmetry fluctuated over the span of a few weeks in subjects with MDD as compared with normal controls (Debener et al., 2000). This finding suggests that the increased alpha band power in MDD might best be viewed as indicating a broadly distributed, shifting state of brain dysregulation rather than a fixed, localized abnormality (Fingelkurts et al., 2006; Park et al., 2007).

Several studies have examined subjects during task activation, and these have yielded more consistent findings. Henriques and Davidson (1997) compared alpha power in depressed subjects with controls during verbal and spatial tasks. They found that MDD subjects had a deficit in spatial task performance that was associated with a deficit in modulation of right hemispheric posterior alpha power. Manna et al. (2010) reported poor alpha modulation in left and right hemispheres on verbal and spatial tasks, respectively, when comparing anxious and non-anxious depressed subjects. Most recently, Seagrave et al. (2010) found that subjects with MDD were less able to desynchronize alpha activity over the left hemisphere during a working memory task than were normal controls. In a large study of subjects with MDD, Leuchter et al. (2012) recently showed significant and widespread increases in resting brain functional connectivity using qEEG coherence. Increases in connectivity were seen across all frequency bands, but most notably in the alpha band. Taken together, these studies indicate a lack of normal modulation of brain function in individuals with MDD.

The inability to modulate regional oscillatory activity, particularly in the alpha frequency band, in response to shifts in the environment or task demands may represent a fundamental neurophysiologic defect in MDD. Integration of brain activity across greater distances is coordinated by lower frequency (e.g., alpha or theta) activity, while shorter-distance coordination is coordinated by higher frequency (e.g., beta and gamma, or 12–20 Hz and 20–40 Hz activity, respectively) (Jann et al., 2009; Britz et al., 2010; Sadaghiani et al., 2010). Synchronized alpha oscillations in particular play a key role in global top-down control of brain cognitive processes that have been shown to be disturbed in MDD (Klimesch et al., 2007; Sadaghiani et al., 2010). Synchronization of oscillatory activity also may reflect, and be linked, to disturbances in central serotonergic tone in MDD (Epstein et al., 2011). Serotonergic projections from the medial septal region dampen theta oscillatory synchrony in the hippocampus (Mu and Han, 2010), while those from the raphe to specific thalamic nuclei modulate alpha synchrony (Kudina et al., 2004). Rhythmic oscillatory activity in qEEG has been shown to be modulated by antidepressant medication (Feige et al., 2005; Dzirasa et al., 2010; Mu and Han, 2010).

Another finding seen consistently across studies of subjects with MDD is reduced rCBF broadly over the prefrontal cortex (Awata et al., 1988; Videbech, 2000; Cintia, 2005). The defect in modulation of alpha power and decreased rCBF appear to be related phenomena, both being associated with severity of depressive symptoms. The degree of decrease in cortical rCBF is correlated with symptom severity (Awata et al., 1988; Mayberg, 1994; Galynker et al., 1998), with the decrease most pronounced in subjects with cognitive impairment associated with their depression (Dolan et al., 1994; Teneback et al., 1999). Similarly, the severity of psychomotor retardation in MDD was found to be correlated with the power of low frequency EEG, including in the lower alpha band (Nieber and Schlegel, 1992). The ability to synchronize and desynchronize qEEG oscillations across the frequency spectrum subserves a range of vital functions within brain networks, from regulation of neurotransmitters to cerebral blood flow. Dysregulation of cerebral oscillatory activity therefore may represent the pathophysiologic link between disturbances in monoaminergic neurotransmission, cerebral perfusion, and brain network dysfunction in MDD (Leuchter et al., 2012).

In considering disturbed brain oscillatory activity in MDD, it is important to note that thalamocortical dysrhythmia may represent either a “top-down” or “bottom-up” process (that is, primarily arising from disturbed cortical or thalamic function) (Llinás et al., 1999). Resonance in the alpha or theta frequency bands is maintained by loops of cells in the cortex and thalamic nuclei that function as part of thalamocortical circuits. Persistent burst patterns of cell firing in thalamic nuclei essentially lock in the resonant frequency through their interaction with the cortical cell population. In Parkinson's Disease and other illnesses of primarily striatal origin, the dysrhythmia may originate primarily in the thalamus, thus representing a bottom-up phenomenon; in the epilepsies, the mechanism may be more top-down in origin, the result of aberrant cortical input (Llinás et al., 1999; Schulman et al., 2011). The essential pathophysiology of MDD remains unknown, and the illness may arise from either “end” of the resonance loop or as the result of an interaction among different cell populations. The neurophysiologic manifestations of the dysrhythmia, however, may be indistinguishable between bottom-up and top-down situations (Llinás et al., 1999). And importantly, from the standpoint of therapeutic effects of rTMS, exogenous input into any portion of a resonance loop may affect the function of all components of the thalamocortical circuit, leading to amelioration of symptoms in a range of illnesses marked by dysrhythmia (Llinás et al., 1999; Jeanmonod et al., 2003; Fuggetta and Noh, 2012).

## Effects of rTMS on motor systems

Most of what is known regarding the effects of magnetic stimulation on brain function has been learned from the effects of rTMS on motor systems. While these effects are critically dependent upon a variety of stimulation parameters, including neuroanatomical target, pulse repetition frequency, waveform type, and field strength, among other factors, the effects on the motor system of adjusting rTMS stimulus parameters are immediately detectable. In addition, the neurophysiologic basis of effects on the motor system is relatively well understood because generation of MEPs may be sufficient to explain the effects of magnetic pulses on the motor cortex (Zarkowski et al., 2006). For example, low frequency stimulation over the primary motor cortex decreases motor excitability, whereas high frequency stimulation increases excitability (Fitzgerald et al., 2006). The waveform of the magnetic pulse also in part helps to determine its effect. rTMS using monophasic rectangular pulses appears to activate a relatively homogeneous population of neurons and its effects more readily summate than biphasic rTMS, so that it has a stronger short-term effect on motor cortical excitability than biphasic rTMS. In contrast, biphasic rTMS activates a range of both excitatory and inhibitory neurons and appears to exert a broader range of effects than monophasic rTMS of similar amplitude (Arai et al., 2005; Sommer et al., 2006; Lefaucheur, 2009).

## The effects of rTMS on mood systems

While focal, high field strength stimulation over particular areas of the motor strip affects contralateral limb movements, it remains unclear what stimulation procedure is optimal to produce consistent therapeutic effects of rTMS on mood. Although high frequency (10 Hz) focal stimulation of the left DLPFC is the most commonly used approach for the treatment of MDD (Slotema et al., 2010), focal stimulation at high frequency in the left and low frequency (1 Hz) in the right DLPFC regions both have been reported to be of significant benefit in relieving symptoms of depression (Gross et al., 2007). It is unknown whether different foci of stimulation, simultaneous stimulation of multiple targets, or broader general stimulation may be of similar or greater therapeutic benefit because these approaches have not been studied systematically.

In contrast to the effects of rTMS on the motor system, its effects in ameliorating symptoms of MDD commonly emerge only over days or weeks of continued treatment sessions (Higgins and George, 2009), so that systematic investigation of the many different stimulus parameters is challenging. The processes by which rTMS yields motor results may be distinct from those operative in using rTMS to treat depression. The laminar structure of motor cortex and the vertical integration of the central motor system are fundamentally different from the organization of brain systems regulating mood. Mood regulatory pathways more diffusely involve multiple areas of dorsolateral, medial orbital, and medial prefrontal cortex, areas that have rich reciprocal interconnections with each other and with deep gray matter structures. The networks subserving mood regulation are not as well defined as those involved in motor function and are more heterogeneous, with patterns of aberrant regional metabolism in MDD varying across individuals (Shafi et al., 2012). Synchronized oscillations play a prominent role in binding together the brain networks that regulate both mood and motor activity. The nature of coordinated oscillatory activity in the motor system, however, may be fundamentally different from that in other brain networks in terms of the frequency bands that play a prominent role as well as the structures that are bound together by synchronous activity (van Wijk et al., 2012). There may be limitations to the extent to which stimulus parameters that have immediate effects on the motor strip (M1) produce optimal therapeutic effects in a different region (such as DLPFC) for the treatment of MDD (Rossi et al., 2009; Hoogendam et al., 2010).

This problem of identifying optimal parameters for treatment of MDD is further compounded by the fact that the MOA underlying the effects of rTMS on mood and thinking in MDD are incompletely understood (Pascual-Leone et al., 2000; Fecteau et al., 2006; Rossi et al., 2009). While phenomena such as muscle contraction following motor strip stimulation or visual phosphenes following stimulation of occipital cortex or retina can be explained through depolarization and neuronal firing, the origins of more complex phenomena such as altering function of cognitive or mood regulating systems is more challenging. It has been proposed that rTMS can produce a functional “virtual lesion” in underlying cortex, temporarily disrupting cognitive processes reliant on that region (Pascual-Leone et al., 2000). Some aspects of the effects of rTMS on the motor system, thinking, and memory may be pertinent to understanding the therapeutic benefit of rTMS on mood. The virtual lesion concept may help explain the clinical effectiveness observed with “slow rTMS” (1 Hz or lower frequencies) administered to the right DLPFC. Increased relative cerebral blood flow and cortical excitability in this brain region are thought to contribute to the symptoms of MDD, and the inhibitory effects of low frequency rTMS may decrease right DLPFC activity, leading to clinical improvement (Speer et al., 2000; Kito et al., 2008; Rossini et al., 2010). Conversely, the left DLPFC region has been reported to have low relative blood flow and decreased cortical excitability in MDD, and “fast rTMS” (e.g., 10 Hz) has been suggested to contribute to a lasting *elevation* in activity in this target region (Rossini et al., 2010).

## Cellular and neurochemical effects of rTMS in MDD

Evidence indicates that the MOA of rTMS on depressed mood may be understood in part through its ability to create enduring effects on synaptic signaling. One line of research demonstrates that rTMS has lasting effects on cortical plasticity (O'Reardon et al., 2007; Cheeran et al., 2008; Gentner et al., 2008; George et al., 2010; Oberman et al., 2010; Stagg et al., 2010; Freitas et al., 2011). Neuroimaging studies have shown that, after a series of rTMS treatments, activity is persistently increased not only in DLPFC, the neuroanatomic target of stimulation, but also in subgenual anterior cingulate and basal ganglia regions, areas that are synaptically connected to DLPFC but are not believed to be stimulated directly because the magnetic field strength at that depth is insufficient to trigger depolarization (Speer et al., 2000; Loo et al., 2003; Kito et al., 2008). Given that multiple regions have been implicated in the pathophysiology of depression (Boynton and Olson, 1990), neuroplasticity involving multiple brain regions linked through cortical-subcortical networks may underlie such persistence of effect (Peinemann et al., 2004; Boggio et al., 2011; Di Lazzaro et al., 2011; Freitas et al., 2011; Iezzi et al., 2011; Pascual-Leone et al., 2011; Song et al., 2011; Nardone et al., 2012).

A second line of research demonstrates that rTMS also has lasting effects on monoaminergic neurotransmission, including alterations in the capacity for serotonin synthesis from tryptophan (Sibon et al., 2007), the release of dopamine (Strafella et al., 2001; Pogarell et al., 2006), and the expression of frontal and striatal adrenergic receptors (Ben-Shachar et al., 1999). There appears to be a link between certain shifts in regional brain activity and serotonergic tone in particular. Baeken et al. (2011) examined the relationship between rTMS and regional postsynaptic 5-HT(2A) receptor binding indices. They found that improvement following left sided 10 Hz rTMS treatment correlated positively with changes in 5-HT(2A) receptor binding indices in the DLPFC bilaterally and negatively with right hippocampal binding. Several small case series have implicated genetic polymorphisms that affect serotonin transporter expression, the 5-HT(1A) receptor, or BDNF in modulating the likelihood of response to rTMS, but results have been inconsistent (Zanardi et al., 2007; Bocchio-Chiavetto et al., 2008; Malaguti et al., 2011). In one study, rapid tryptophan depletion did not lead to reemergence of depressive symptoms in adults who had responded to a course of rTMS (O'Reardon et al., 2007), suggesting that the therapeutic effects of rTMS do not depend critically upon central serotonergic tone.

Effects on neuroendocrine measures also have been reported in animals (Keck et al., 2001; Hedges et al., 2003; Kito et al., 2010) and in humans undergoing rTMS (Pridmore, 1999; Cohrs et al., 2001; Zwanzger et al., 2003), and hippocampal neurogenesis has been reported in animals (Czeh et al., 2002). A cautionary note about interpreting these animal studies has been sounded by Lisanby and Belmaker (2000): while therapeutic rTMS in humans tends to involve stimulation of a spatially-limited portion of brain tissue, most if not all of the animal brain is exposed to high levels of the magnetic field in these experimental paradigms, due to the different size of animals and the physics of generating magnetic fields.

## Cerebral oscillatory activity and the mechanism of action of rTMS in MDD

Observations regarding changes in cerebral neurotransmitter levels and cortical plasticity and blood flow patterns (Paus and Barrett, 2004) indicate that the MOA of rTMS is best understood at the level of the brain as an organ system, with neurochemical and neuroplastic changes seen in regions that are far removed from the site of stimulation (Pogarell et al., 2006; Sibon et al., 2007; Cho and Strafella, 2009). In a study of cerebral blood flow following rTMS administered to DLPFC, Paus et al. (2001) demonstrated that low-frequency rTMS caused decreased blood flow not only in the area being stimulated, but also in other regions including the anterior cingulate cortex; conversely, high-frequency rTMS caused increases blood flow in the same regions. These findings indicate that rTMS modulates activity through distributed brain networks.

These findings beg the question, however, as to how administration of repetitive rhythmic magnetic pulses induces neuroplastic changes in these networks. Evidence indicates that rTMS may be linked to alterations in neuroplasticity, neurotransmission, and blood flow through the effect of rTMS on cerebral oscillatory activity. The most immediate effect of rTMS on brain function is alteration of the oscillations of underlying brain tissue as measured with the electroencephalogram (EEG). High-frequency stimulation (>5 Hz) leads to an immediate synchronization of EEG activity in the alpha and beta bands, consistent with entrainment of oscillatory activity (Paus et al., 2001; Klimesch et al., 2003; Fuggetta et al., 2005, 2008; Brignani et al., 2008; Hamidi et al., 2009; Johnson et al., 2010; Thut et al., 2011); entrainment following stimulation also has been reported in the delta and theta bands (Fuggetta and Noh, 2012). This entrainment has been reflected in findings of increased power (Fuggetta et al., 2005; Brignani et al., 2008; Fuggetta and Noh, 2012) and coherence (Fuggetta et al., 2008) particularly in the alpha frequency band as assessed with EEG.

**Figure 1:**
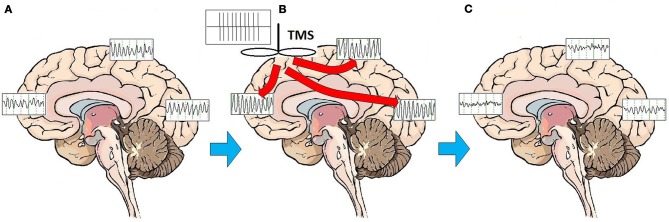
**Effects of rTMS stimulation on brain function.** On average, patients with MDD exhibit a broad pattern of highly synchronous theta and alpha activity over most brain regions **(A)**. rTMS administered as a train of high amplitude pulses at a frequency of 10 Hz entrains brain oscillatory activity to the frequency of stimulation, for the duration of the stimulation period **(B)**. Multiple treatments over time may have the effect of resetting cortical oscillators. Once oscillators are reset, regionally-specific endogenous rhythms of the brain may reemerge. These consist of beta and gamma activity in the frontal cortex, beta in the parietal cortex, and alpha in the occipital cortex **(C)**.

This pronounced and immediate entrainment of oscillatory activity suggests that the modulatory activity of rTMS on brain structures and circuits may be accomplished by altering the frequency and patterns of brainwave oscillatory activity. A series of studies has demonstrated that high frequency rTMS leads to alterations of cortical oscillations at the site of stimulation, and frequently at more distant areas as well, consistent with linkage of brain regions through corticocortical and thalamocortical loops (Fuggetta et al., 2005, 2008; Brignani et al., 2008; Hamidi et al., 2009; Thut and Miniussi, 2009; Thut and Pascual-Leone, 2010). Although the effect of high-frequency rTMS is to elicit local cortical oscillations predominantly at the frequency of stimulation for the duration of stimulation (Paus et al., 2001; Rosanova et al., 2009), rTMS does not broadly entrain cortical rhythms to the stimulation frequency on a sustained basis. Once stimulation has ceased, rTMS appears primarily to enhance or “bias” the natural rhythms of underlying cortex (Hamidi et al., 2009; Johnson et al., 2010). In fact, outside of the target area of immediate cortical stimulation, rTMS evokes alpha activity in the occipital cortex, beta activity in the parietal cortex, and beta/gamma activity in the frontal cortex (Rosanova et al., 2009). In multiple experimental paradigms, high frequency (10 Hz) rTMS pulses appear to trigger and reset oscillatory mechanisms (Paus et al., 2001; Fuggetta et al., 2005, 2008; Brignani et al., 2008), marked by a so-called “event related synchronization” (ERS) at the frequency of stimulation in the area that has been stimulated, followed rapidly by an “event related desynchronization” (ERD) and re-emergence of endogenous rhythms (Jäncke et al., 2006; Zarkowski et al., 2006; Brignani et al., 2008; Sauseng et al., 2008). One study recently suggested that low frequency (1 or 5 Hz) stimulation may facilitate emergence of local endogenous rhythms by disrupting persistent low frequency thalamocortical resonance phenomena (Fuggetta and Noh, 2012). Regardless of the frequency of stimulation, enhancement of the reemergence endogenous local cortical and thalamocortical rhythms may be central to the MOA of rTMS stimulation. rTMS may act through entraining oscillations to the frequency of stimulation, thus resetting cortical and thalamocortical oscillators and facilitating the reemergence of normal, intrinsic oscillatory activity (Paus et al., 2001; Fuggetta et al., 2005, 2008) (Figure 1).

## New approaches to resetting thalmocortical oscillators

It may be possible to enhance the effectiveness of rTMS as a treatment for depression through application of our current understanding of the MOA of rTMS. MDD is a brain disorder marked by widespread dysregulation of oscillatory synchrony (Leuchter et al., 2012), and through resetting cortical and thalamocortical oscillators, rTMS may ameliorate the symptoms of MDD (Paus et al., 2001; Fuggetta and Noh, 2012). Evidence suggests that the effectiveness of rTMS in resetting oscillators may be increased through: (1) synchronization of rTMS to the individual alpha frequency (IAF), (2) modification of field strength and the waveform of stimulation, and (3) broadening the area of stimulation. Each of these strategies is discussed in greater detail below.

## Synchronization of rTMS to the individual alpha frequency

Although a wide range of stimulation frequencies has been shown to modulate brain function (Thut and Pascual-Leone, 2010), therapeutic application of rTMS commonly has focused on stimulation in the alpha frequency band (Thut et al., 2011; Veniero et al., 2011). This is consistent with current understanding of the role of alpha oscillatory activity, which now is conceptualized as playing a key role in maintaining coordinated activity among cortical areas and between the cortex and subcortical gray matter structures (e.g., thalamus) (Buzsáki and Draguhn, 2004). There is evidence that endogenous activity in the alpha band in particular may exert top-down control over broadly distributed brain regions, allowing selective activation of brain areas with consequent alterations in brain circuit function (Klimesch et al., 2007; Benedek et al., 2011). Alpha frequency oscillations appear to be particularly well suited to coordinating activities over distance in the brain, in part because of the influence of thalamic pacemakers (Buzsáki and Draguhn, 2004). Upper alpha band power (10–12 Hz), as measured with qEEG, is involved in modulating connections among the dorsal anterior cingulate cortex, anterior insula, anterior prefrontal cortex and thalamus (Sadaghiani et al., 2010). Several of these brain areas are involved both in cognition and in regulation of mood. Alpha activity, particularly in the lower band (8–10 Hz), fulfills the function of an inhibitory oscillatory rhythm. A highly synchronous alpha state can bind brain areas together in a preparatory manner in an ERS prior to their involvement in a cognitive or motor task (Klimesch et al., 2007). This inhibitory synchronized state is preparatory for the task that follows because it facilitates recruitment of specific regions of cortex for actual task involvement. The task execution itself is marked by an ERD, or attenuation of synchronous alpha activity and the emergence of largely asynchronous beta and gamma activity (Klimesch et al., 2003; Brignani et al., 2008). Brain areas that are in a highly synchronous alpha state frequently show high alpha band power and low regional cerebral blood flow (rCBF), and following an ERD show low alpha power and higher rCBF (Feige et al., 2005). The effects of rTMS pulses on motor and cognitive functions are complex, with some studies showing detrimental effects and others enhancement effects on task performance. This appears to depend upon several factors including whether the pulses are delivered before, during, or after the task (Hamilton and Pascual-Leone, 1998; Wassermann et al., 1999; Evers et al., 2001; Sparing et al., 2001; Klimesch et al., 2003; Brignani et al., 2008; Hamidi et al., 2009).

Klimesch et al. (2003) have proposed that one key factor determining whether exposure to rTMS enhances or degrades task performance is the relationship of the frequency of rTMS stimulation to the subjects' IAF. This group demonstrated rTMS delivered at a subject's IAF plus 1 Hz (IAF + 1) enhanced performance on a mental rotation task compared with pulses at a lower individualized frequency (IAF − 3) or at a fixed, higher (20 Hz) stimulation frequency. Similarly, Hamidi et al. (2009) observed that among subjects performing cognitive tasks in association with 10 Hz rTMS, those with higher IAF tended to have higher task performance accuracy. A central role for modulation of IAF in the MOA of rTMS is consistent with the increased understanding of the importance of alpha frequency band activity in regulating brain functions. Higher mean IAF is associated with greater rCBF at rest, greater preparedness for external input, and greater neural efficiency in task performance (Jann et al., 2010). In addition, higher power and broadly synchronized alpha activity is associated with lower regional blood flow (Feige et al., 2005). Taken as a whole, these findings indicate an association between aberrant alpha activity, rCBF, and MDD severity, such that the aberrant alpha activity may constitute one of the functional underpinnings of network dysregulation in MDD.

Stimulation at an individual's intrinsic alpha frequency can lead to activation or inhibition of a brain region, and “tuning” of rTMS to the IAF may enhance effectiveness of the procedure. rTMS delivered at the IAF may be particularly effective at resetting dysfunctional cortical oscillators, which may lead to increased cerebral blood flow and therapeutic benefit in MDD. No published study to date has systematically examined the effects of rTMS pulses delivered at the actual IAF and a spread of frequencies narrowly higher and lower the IAF; such studies would be necessary to establish definitely a central role for IAF as a target for the frequency of rTMS stimulation. Although no treatment trials have specifically utilized treatment at the IAF, two trials in schizophrenia have employed IAF stimulation. In the first of these two trials, TMS delivered at the IAF demonstrated a significantly larger therapeutic effect than TMS at other frequencies (29.6% reduction in negative symptoms, vs. <9% for other stimulation settings, *p* = 0.007) (Jin et al., 2006). In the later trial (Jin et al., 2012) using sham-controlled conditions, TMS tuned to the IAF produced a significantly greater clinical effect than sham, regardless of whether delivered over frontal or parietal locations. While one trial in MDD used information about the IAF to guide rTMS stimulation frequency choice (Arns et al., 2010), the investigators compared personalized IAF + 1 stimulation [as proposed by Klimesch et al. (2003)] with stimulation at 10 Hz, but did not directly compare stimulation *at* IAF with stimulation at other frequencies. They reported that stimulation at IAF + 1 was not superior to stimulation at a fixed frequency of 10 HZ, and that for lower IAF (e.g., 8 Hz), the individualized stimulation frequency may have been less effective. It is important to note that this report is based upon a small open-label case series, and that the treatment was administered at IAF + 1 and not IAF. Further research would be necessary to evaluate the therapeutic effectiveness of rTMS delivered at the IAF.

## Modification of field strength and the waveform of stimulation

In addition to the frequency at which rTMS is delivered, the field strength and waveform used for stimulation are important treatment variables. rTMS commonly is delivered utilizing a train of high field strength (1.5–4 Tesla) pulses administered with an electromagnetic coil to a discrete brain region. Recent research has examined the use of different coil sizes and configurations, and devices that perform stimulation using multiple coils, to perform more intense, focused, or deeper brain stimulation. Research on the use of different coils is ongoing (Deng et al., 2013), and a detailed discussion is beyond the scope of this review.

The paradigm of intense focal stimulation is predicated in part on the precedent of motor system stimulation, and in part on the pathophysiology of MDD in which there is hypometabolism of the left DLPFC. It is not clear, however, that high field strength pulses administered to a specific location is necessary to obtain the therapeutic effects of rTMS in the treatment of MDD. Recent research suggests that the neurophysiologic effects of stimulation can be achieved with low electromagnetic field potential. Subthreshold rTMS (i.e., delivered without depolarizing neurons) has been shown to affect alpha band power and coherence more than superthreshold rTMS (Fuggetta et al., 2008). Furthermore, extremely weak magnetic fields have been demonstrated to have significant effects on waking EEG, most notably in the alpha band (Cook et al., 2005, 2009). Low magnetic field strengths also have been shown to affect cerebral glucose metabolism (Volkow et al., 2010). These findings are consistent with recent work indicating that low strength fields can strongly entrain action potentials of cortical neurons through ephaptic coupling (Anastassiou et al., 2011).

Weak sinusoidal waveforms have not been as extensively studied as have monophasic or other biphasic waveforms, but research indicates that they may be effective in altering brain function. In an animal model, a weak sinusoidal magnetic field in conjunction with a static field had significant effects on reducing EEG power (Vorobyov et al., 1998). Sinusoidal waveform magnetic stimulation produces sinusoidal electrical fields (EFs) in the brain. Weak sinusoidal EFs have potent effects in entrainment of cortical oscillations both in animals (Ozen et al., 2010) and *in vitro* (Francis et al., 2003; Fröhlich and McCormick, 2010; Anastassiou et al., 2011), as well as in modulating and biasing endogenous cortical oscillations (Reato et al., 2010), even at subthreshold levels that do not lead to neuronal depolarization and action potential discharges.

The effects of low magnetic field stimulation both on EEG and metabolism suggest that it may hold therapeutic promise in MDD. The potential usefulness of these low field intensities in treatment is supported by reports that low magnetic field strengths may improve mood in patients with treatment-resistant depression (Rohan et al., 2004; Carlezon et al., 2005; Rokni-Yazdi et al., 2007; Martiny et al., 2010).

Application of transcranial alternating current stimulation (tACS) also supports the concept that low energy sinusoidal waveforms may have therapeutic benefit. In this technique, low levels of sinusoidal electrical current are administered across the skull in order to alter brain activity in a large region of tissue without evoking a seizure but with demonstrable behavioral and neurophysiologic effects (Paulus, 2011). Applying sinusoidal slow oscillating transcranial potentials (0.75 Hz) to healthy subjects during early sleep, Marshall et al. (2006) demonstrated enhancement of declarative memory and an associated increase in slow wave sleep, endogenous cortical slow oscillations, and slow spindle activity in the frontal cortex. This work demonstrates both entrainment of cerebral oscillatory activity in humans using low-field potentials and its functional effects. Using a brief (≤10 min) application of 10 Hz tACS in healthy adults, administered at low current (<0.5 mA) over the primary motor cortex, researchers (Antal et al., 2008) found significant improvements in the acquisition and early consolidation phase of implicit motor learning in a serial reaction time task. Recent behavioral work with tACS (at a theta-band 6.5 Hz frequency) applied to the left DLPFC led to a riskier decision-making pattern compared with right DLPFC or sham stimulation (Sela et al., 2012). In an examination of neurophysiologic effects, Zaehle et al. (2010) demonstrated the ability of tACS to alter the EEG in healthy adults. In their work, tACS was applied over the occipital regions, using a frequency personalized to the IAF of each subject. They found that tACS elevated the endogenous alpha power in the parieto-central region, whereas sham stimulation did not. Frequencies other than IAF were not examined. Their work demonstrated that tACS could produce entrainment of brain rhythms, but these projects did not study clinical or behavioral effects in depressed population. This line of work supports the value of further research into the use of low energy sinusoidal waveform stimulation of the brain in MDD.

There are other forms of low-intensity brain stimulation, including “cranial electrotherapy stimulation” (CES). The physiologic effects of this and other methods of stimulation have not been the subject of systematic study. The extent to which CES current enters the brain and its possible effects on brain function and mood are not well documented or understood.

## Broadening the area of stimulation

**Figure 2:**
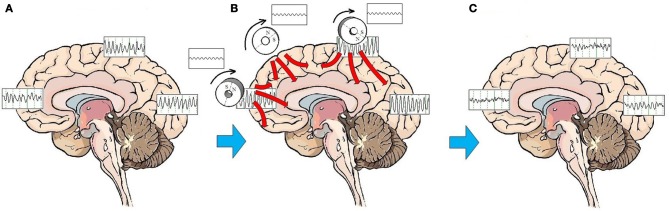
**Effects of sTMS stimulation on brain function.** On average, patients with MDD exhibit a pattern of highly synchronous theta and alpha activity seen broadly over most brain regions **(A)**. The low-amplitude sinusoidal stimulation provided by the neodymium magnets rotating at the IAF entrains brain oscillatory activity to the frequency of stimulation for the duration of the stimulation period **(B)**. Like the high amplitude pulses of rTMS, multiple treatments of subthreshold sinusoidal stimulation may have the effect of resetting cortical oscillators. Once oscillators are reset, regionally-specific endogenous rhythms of the brain may reemerge. These consist of beta and gamma activity in the frontal cortex, beta in the parietal cortex, and alpha in the occipital cortex **(C)**.

Entrainment of cerebral oscillatory activity appears to be an essential step in resetting cortical oscillators. The customary approach to rTMS is to apply stimulation to a single target, most commonly over the right or left DLPFC. As discussed above, stimulation to a single target area can have neurophysiologic and neuroplastic effects in distant areas connected through brain networks. An alternative to targeting a specific mood regulating area is to stimulate the brain broadly. A procedure to administer low-field magnetic stimulation to large areas of the brain has been described by Phillips and Jin (2012). This procedure utilizes a device that contains three cylindrical neodymium magnets positioned close to the scalp distributed along the midline from the prefrontal to the parietal region. The magnets are rotated at a programmable frequency, generating a sinusoidal magnetic waveform that imparts stimulation along the parasagittal line (Figure 2). A preliminary feasibility study of 45 subjects treated with low-field sinusoidal magnetic stimulation synchronized to the IAF (synchronized TMS, or sTMS) showed that 44.8 % of subjects responded to the treatment, significantly greater than the proportion responding to sham treatment (Phillips and Jin, in submission). It has not yet been demonstrated that sTMS is superior to rTMS, and a multi-center double-blind controlled clinical trial to investigate the safety and effectiveness of sinusoidal magnetic fields delivered at the patient's IAF to treat MDD is currently being conducted by NeoSync, Inc (ClinicalTrials.gov Identifier NCT01370733).

## Conclusion

Converging lines of evidence indicate that MDD is linked to alterations in cerebral blood flow, metabolism, and regulation of neuronal oscillatory activity. These abnormalities in brain physiology are reflected in the severity of mood disturbance, neurovegetative symptoms, and cognitive dysfunction. Disturbed oscillatory activity is most clearly evident in the alpha frequency band, where qEEG detects disturbances in power and coherence.

The disturbance in regulation of alpha activity is consistent with the effectiveness of alpha frequency rTMS in the treatment of MDD. rTMS administered in the alpha frequency band promotes immediate ERS followed by ERD. Over time, this repetitive entrainment of cerebral oscillators may represent the MOA for rTMS in the treatment of MDD. Entrainment of cerebral oscillations by exogenous stimulation serves to reset cortical oscillators, possibly enhancing neuroplasticity, normalization of cerebral blood flow, and amelioration of depressive symptoms.

Specific modifications of the rTMS methods that are currently employed in clinical practice could lead to enhanced efficacy of the technique for treatment of depression. First, administration of alpha frequency rTMS that is synchronized to the patient's IAF should be evaluated as an alternative to fixed-frequency 10 Hz rTMS, a widely employed setting and the one which has been approved by the US Food and Drug Administration for therapeutic use. Data suggest that ERS and resetting of cortical oscillators may be most effectively achieved by stimulation at the oscillatory frequency that is specific for each individual. Second, subthreshold sinusoidal waveform magnetic stimulation may be as effective, if not more effective, at resetting cortical oscillators as high-field electromagnetic stimulation. Low field stimulation may be not only be as effective, but could be better tolerated by patients. Third, because alpha dysregulation in MDD is widespread across brain regions, it is possible that stimulation administered to the brain more broadly, may be at least as effective as stimulation delivered to the conventional DLPFC target in ameliorating the symptoms of MDD. Future studies of the effectiveness of brain stimulation therapies should examine not only amelioration of depressive symptoms, but also improvements in cognition and functional status, as cognitive and functional deficits are common features of the syndrome of MDD. Tailoring of the method of delivery of rTMS to improve the effectiveness with which cortical oscillators are reset may more effectively normalize neurophysiologic abnormalities in subject with depression, and enhance the effectiveness of this treatment for MDD.

### Conflict of interest statement

Drs. Phillips and Jin are currently employed by NeoSync, Inc., a company dedicated to the development of synchronized TMS (sTMS) technology. They are the Chief Technology Officers, and founders of the company with a significant equity interest. They are listed as inventors on patent applications for sTMS. Dr. Leuchter is a consultant to NeoSync, Inc. and Covidien, Inc. Dr. Cook has received research support from NeoSync, Inc.
